# Environmental Transformations of Metals in a Hypersaline Mediterranean Agricultural Wetland (Laguna Honda, South Spain)

**DOI:** 10.3390/toxics14090818

**Published:** 2026-09-14

**Authors:** Antonio Medina-Ruiz, Juan Jiménez-Millán, Isabel Abad, Rosario Jiménez-Espinosa

**Affiliations:** Departmento de Geología and Centro de Estudios Avanzados en Ciencias de la Tierra, Energía y Medio Ambiente (CEACTEMA), Universidad de Jaén, Campus Las Lagunillas, 23071 Jaén, Spain; medina@ujaen.es (A.M.-R.); miabad@ujaen.es (I.A.); respino@ujaen.es (R.J.-E.)

**Keywords:** heavy metals, statistical factor analysis, sulfides, mercury, copper, gold, HRTEM

## Abstract

This study investigated the biogeochemical processes governing heavy metal sequestration in Laguna Honda, a hypersaline Mediterranean wetland affected by intensive olive cultivation located in the Guadalquivir Basin River (S Spain). A network of sediment samples was analyzed from the wetland using X-ray fluorescence, ICP-MS, and high-resolution electron microscopy (FESEM/HRTEM). Geochemical data were treated with statistical factor analysis. Sediments show significant enrichment of Cu, Hg, and Au due to anthropogenic agrochemical treatments. Mineralogical characterization revealed authigenic pyrite framboids and metallic sulfide nanoparticles (CuS and HgS) within organic-rich sediments, where sequestration is primarily probably driven by the metabolic activity of sulfate-reducing microorganisms (SRMs) under reducing conditions. Additionally, Au enrichment is attributed to mechanical transport and coagulation of pesticide-derived nanoparticles, exhibiting a distinct geochemical correlation with rare earth elements (REEs). We concluded that hypersaline wetlands act as critical environmental sinks, where microbial activity, high organic matter content, and detrital transportation interact to facilitate the immobilization and stabilization of toxic trace elements through the precipitation of low-solubility sulfide phases (Cu and Hg) and the mechanical accumulation of nanoparticles (Au).

## 1. Introduction

Wetlands are important environmental systems; they commonly have legal protection due to their regulation of surface and groundwater, accumulation of eroded materials as sediments, and preservation of wildlife habitats in terrestrial systems [1,2,3]. However, in the Mediterranean area, wetlands are frequently located in the topographic depressions of crop areas, which accumulate significant amounts of nutrients and residual toxic substances (such as heavy metals) used in agricultural practices [2,4,5]. The concentration of decaying organic matter in the presence of important microorganism communities and the presence of areas with seasonal submersion promote variations in redox conditions, which control the fixation or mobility of metals [3,6,7,8]. This makes wetlands sensitive systems to the sinking of trace elements [9,10].

Cu and Hg are common sediment pollutants; they can produce intense environmental degradation in wetland crop areas as a result of anthropogenic activities. Cu compounds are antimicrobial agents and biocides frequently used for crop intensification, leading to an increase in agricultural productivity [11,12,13]. The extensive application of Cu as low-cost and highly effective fungicides and antimicrobial compounds [14] has resulted in the accumulation of Cu in different crop soils and groundwater. Ballabio et al. [15] revealed that Cu contents in agricultural soils are clearly higher than in forest soils due to the intense application of Cu fungicides. The mean European Cu concentration in olive groves (33.5 ppm) is notably higher than the mean contents present in croplands (18.91 ppm).

The long residence time of mercury and its high potential for bioaccumulation [16] favor its omnipresence in natural systems, e.g., [17,18]. This is due to the broad range of anthropogenic emissions associated with industrial and mining pollutant activities and their human use in products such as fungicides and pesticides, which reached a maximum in the 1970s. As a result, Hg fungicides were forbidden in the European Union at the end of the 1970s [19]. However, Hg concentrations above the admissible limits established by the EU can be found for priority substances in natural and constructed wetlands, such as discharges from crops intensely treated with agrochemicals and pesticides, e.g., Albufera Lagoon [20].

Environmental availability of sulfur (S) strongly influences the mobility of metals [7,21,22]. Water SO_4_^2−^ ions are an important source of sulfur in hypersaline wetlands, e.g., [23,24,25,26]. The generation of sulfide ions from sulfate reduction in environments rich in organic matter favors the precipitation of low-solubility metal sulfides, which control environmental metal availability [25,27,28,29,30,31]. Metal fixation via sulfide precipitation in low Eh surface conditions is mainly controlled by the activity of sulfate-reducing microorganisms (SRMs) because the high activation energy of the abiotic reaction inhibits this process [32]. The stability of the precipitated sulfide is influenced by the size, structure, and crystallinity of the mineral [33] and periodic environmental changes. An increase in Eh conditions and higher activity of sulfur-oxidizing microorganisms (SOMs) can produce the oxidation of sulfides and environmental metal release, e.g., [25,34].

The sequestration of metals through crystallization of sulfides requires enough availability of sulfide anions, essentially generated by the activity of SRMs under the environmental conditions of the Earth’s surface [25,35]. Given that the abundance of iron is much higher than other metals with chalcophile characteristics, the precipitation of sulfides is controlled by the formation of iron sulfide phases (greigite, mackinawite, and pyrite). However, the fixation of Cu or Hg, influenced by the precipitation of sulfides, has been reported in contaminated continental-reduced environments, e.g., [3,22].

Agrochemicals are intensively used in Mediterranean olive crops to palliate decreases in productivity as a consequence of climate change, producing negative effects on environmental systems [36]. The use of metal nanoparticles in this type of crop has increased given that it allows controlled liberation of nutrients and pesticides [36,37,38]. More than 1.6 million ha of olive culture land is situated in Andalusia (the south of Spain), contributing to the largest production of olive oil worldwide [39]. High concentrations are located in Jaén and Cordoba provinces in the Guadalquivir River Basin (GRB) [40,41]. Wetlands with important evidence of eutrophication due to the agricultural practices of olive crops are found in the GRB, where significant metal nanoparticle concentrations associated with nutrient and pesticide uses are produced [42,43]. The fate of metals in these wetlands is affected by the transformation of nanoparticles in complex environments. Medina et al. [42] focused on the factors controlling the fate of Au nanoparticles in sediments from Laguna Honda. We aim to clarify the processes involved in the formation of Cu-Hg sulfide nanoparticles, including which metals originate from agrochemicals, in sediments rich in the organic matter of a GRB wetland associated with high-salinity water and olive agriculture.

Iron, copper, and mercury sulfides are homogeneously distributed in sediments rich in organic matter, where aragonite is also frequently present. Previous studies have revealed the presence of copper, gold, and mercury as common sedimentary contaminants that can cause severe environmental degradation in wetlands located in agricultural areas as a result of anthropogenic activities [42,43,44,45,46]. This work aims to improve our understanding of metal behavior in the saline lake environment by integrating the geochemical characterization of wetland sediments with the mineral and biogeochemical processes occurring within it.

## 2. Materials and Methods

### 2.1. Study Area and Materials

Laguna Honda wetland is a karstic morphogenetic system formed by the dissolution of evaporitic materials. It is fed by groundwater and surface water with a floodable potential of around 8.5 ha. The catchment area occupies 96.2 ha. The depth in the wetland progressively increases from the NE to the SW, with a deltaic zone on the northern shore of the lake, which frequently emerges during periods of drought. A depth of 2.5 m is reached in the permanently flooded southern part of the lake. The predominant climatic features of this zone are semi-arid conditions with average temperatures of 12 °C and 30 °C from winter to summer. High-intensity rainfall episodes are concentrated from October to May, alternating with a long dry season [47]. The average annual rainfall is 551 m.

**Figure 1:**
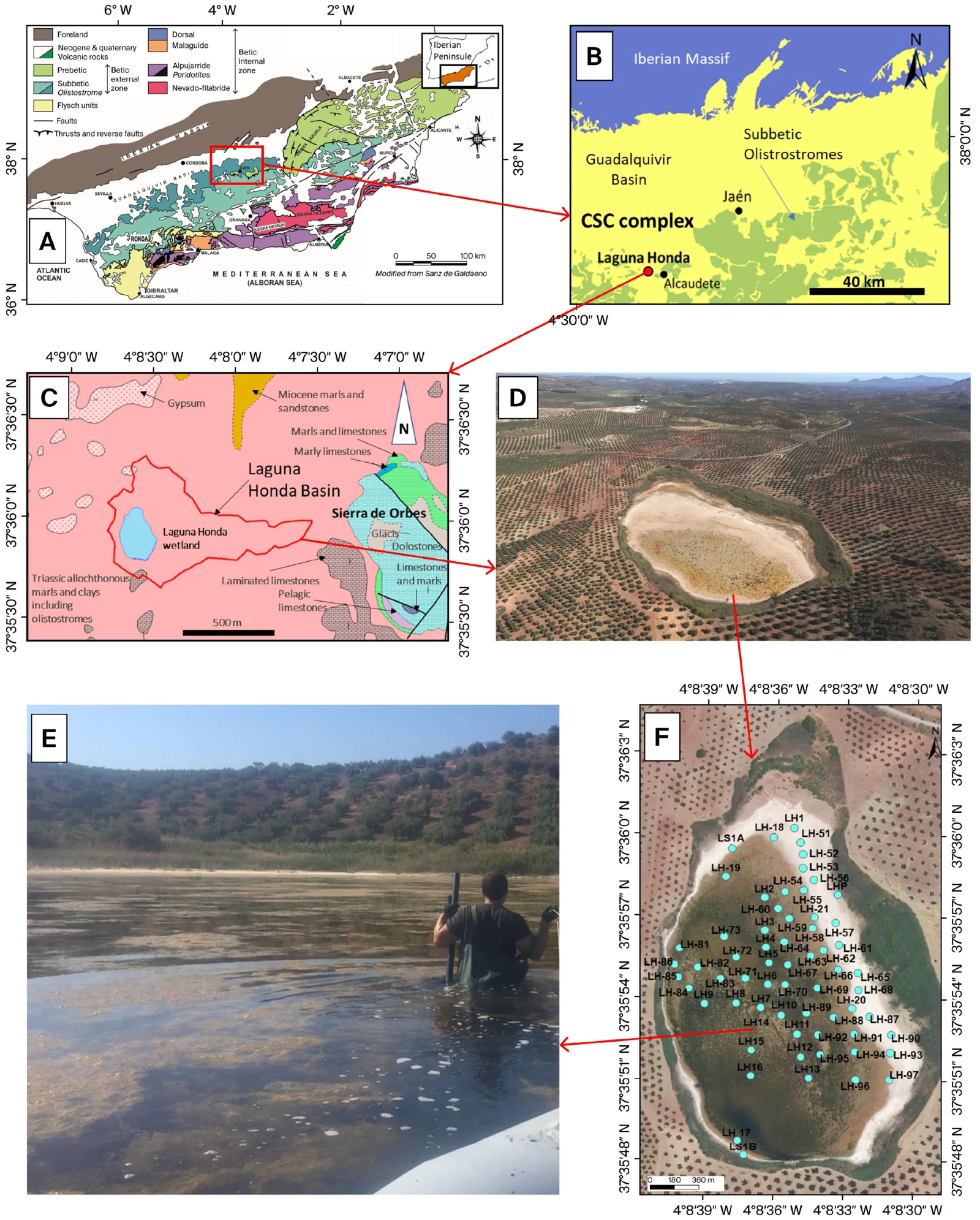
Location of the study area. (**A**) Geographical setting and global geological context of the Betic Cordillera and the study area (modified from Sanz de Galdeano [51]). (**B**) Geological map of the chaotic subbetic complexes (CSCs) near the study area. (**C**) Local geological map of the Laguna Honda area. (**D**) Panoramic view of the Laguna Honda wetland obtained by drone, showing its flooded area, the typical extensive olive groves of the GRB, and the development of algal mats. (**E**) Detail of pale pink floating algal mats. Picture taken by the authors. The person in the picture a member of the author team agreed to participate in the photo (**F**) Sediment sampling points. Modified from Medina et al. [42] under the CC BY 4.0 license, 2024, MDPI.

From a geological point of view, this wetland is in the post-orogenic Guadalquivir River basin close to the External Zone of the Betic Cordillera (Figure 1), specifically one of the chaotic subbetic complexes (CSCs) of the river basin. These complexes are composed of olistoliths at different geological intervals (Triassic, Cretaceous, and Miocene). The materials proceed from the External Betic Zone and were resedimented in the middle Miocene gypsum-rich unit of the Guadalquivir River Basin [48,49,50].

Laguna Honda wetland is surrounded by a monoculture of olives (Figure 1D) on high-slope areas, mostly on the southern shores of the wetland. As a consequence of tillage operations in the olive groves, which permanently maintain bare soil, intense soil degradation processes take place. Moreover, the use of fertilizers for the olives leads to the eutrophication of water, producing seasonal algal blooms (Figure 1E) that colonize the sediments as macrophytes, charophytes, and helophytes (*Ruppia drepanensis*, *Chara galioides*, and *Najas marina*) [52].

The origin and hydrochemistry, vegetation composition, and mineral sediment constituents are characterized in the study of Medina et al. [42,43,44,45,46] and the summary table (Table 1). It is worth highlighting the hypersaline and alkaline characteristics of the waters, which show elevated electrical conductivity, positive oxidation–reduction potential (ORP) values at the surface, and a negative ORP near the bottom of the lake. Regarding microorganisms inhabiting the wetland, Medina et al. [46] revealed that Laguna Honda sediments are characterized by important populations of SRM in deep sediments, promoting sulfide and aragonite precipitation as well as SOMs and other S purple bacteria and bacterial communities resistant to heavy metal stress in shore sediments.

### 2.2. Methods

A network of 54 points was used for sampling (Figure 1F). The cores of sediments on the bottom of the lake were taken from a depth of 0 to 30 cm using PVC tubes sealed with plugs and stored in a portable cooler at T < 5 °C, obtaining a total of 130 samples. All experimental procedures employed to determine the physicochemical parameters of sediments and water, as well as X-ray diffraction for mineralogical characterization, total organic carbon (TOC) analysis, and microbiological characterization, are described in detail in the work of Medina et al. [42,46].

To characterize the presence of sulfides in sediments from the Laguna Honda wetland using electron microscopy (field emission high-resolution scanning electron microscopy, FESEM, and high-resolution transmission electron microscopy, HRTEM), nine representative and regularly spaced shore and central sampling points with high concentrations of Cu and Hg were selected. For the SEM study, sediment samples were mounted on supports and carbon-coated before they were examined using secondary electron imaging (SE) and energy-dispersive X-ray spectroscopy (EDX) analysis to obtain textural and chemical data relative to the mineral composition. In addition, a backscattered electron (BSE) detector was used to complete the characterization of minerals with metallic content. These observations were carried out using a Carl Zeiss Merlin Gemini II FESEM (Carl Zeiss, Oberkochen, Germany) with a resolution of 0.8 nm and five detectors (including one in-lens detector for high resolution) at the CICT (University of Jaén). For nanometric characterization in the TEM, samples were prepared using Ni grid surfaces coated in a formvar resin from the dispersion of finely ground sample particles in alcohol. The TEM study was carried out at the CIC (University of Granada) with a Thermo Fisher Scientific TALOS F200X microscope (Thermo Fisher Scientific, Eindhoven, The Netherlands) operated at 200 kV, equipped with a super-X system (four energy dispersion X-ray EDX detectors) (Eindhoven, The Netherlands); a point-to-point resolution of 0.12 nm was used in the TEM mode and 0.19 nm in the scanning transmission electron microscopy (STEM) mode.

The selected samples were prepared as beads with Philips Pearlx3 for bulk-rock analysis of the major elements in a PANalytical Zetium wavelength-dispersive X-ray fluorescence (XRF) spectrometer (PANalytical, Almelo, The Netherlands) with a maximum power of 4 kW, equipped with an X-ray tube and a Rh anode. Trace elements were analyzed in the same samples using a NexION 300D inductively coupled plasma-mass spectrometer (ICP-MS) (Perking Elmer, Singapore) after HNO_3_^+ ^HF digestion of 100 mg of sample powder in a Teflon-lined vessel at 180 °C and 200 p.s.i. for 30 min, evaporation to dryness, and subsequent dissolution in 100 mL of 4 vol % HNO_3_. These two techniques were performed at the CIC (University of Granada). Certified standards (BR-N, GH, DR-N, UB-N, AGV-N, MAG-1, GS-N, and GA) were used for quantification. The detection limit was below 1 μg/kg; values below this limit were considered 0. The standard deviation was always below 5%. The absence of drift during the Au analysis was verified by measuring two Au standards (5 and 10 ppb, Cole-Parmer) every ten samples. High concentrations of Au and Hg were verified using field emission high-resolution FESEM and HRTEM, which found a significant presence of Au and Hg nanoparticles in sediments. Based on the morphology of nanoparticles, Medina et al. [42] concluded that unusually high concentrations of pollutants are associated with inappropriate application of pesticides. In order to provide information on regional background Cu, Hg, and Au concentrations, two samples of marly sediments surrounding the wetland from the CSCs were taken where Laguna Honda formed.

Statistical relation of variables was evaluated using a factor analysis method with IBM SPSS Statistics Software (version 29) (IBM Corp.; Foster City, CA, USA). Factor analysis was conducted using the principal axis factoring extraction method with Varimax rotation. No prior data transformation was conducted, as the analysis was performed using the correlation matrix, which standardizes the variables. Data adequacy was confirmed via the Kaiser–Meyer–Olkin measure (KMO = 0.85) and Bartlett’s test of sphericity (χ^2^ = 1234.5, *p* < 0.001). Factors were retained based on the Kaiser criterion (eigenvalues > 1). Missing data were handled with pairwise comparisons of 130 samples.

## 3. Results

### 3.1. FESEM-EDX Characterization

**Figure 2:**
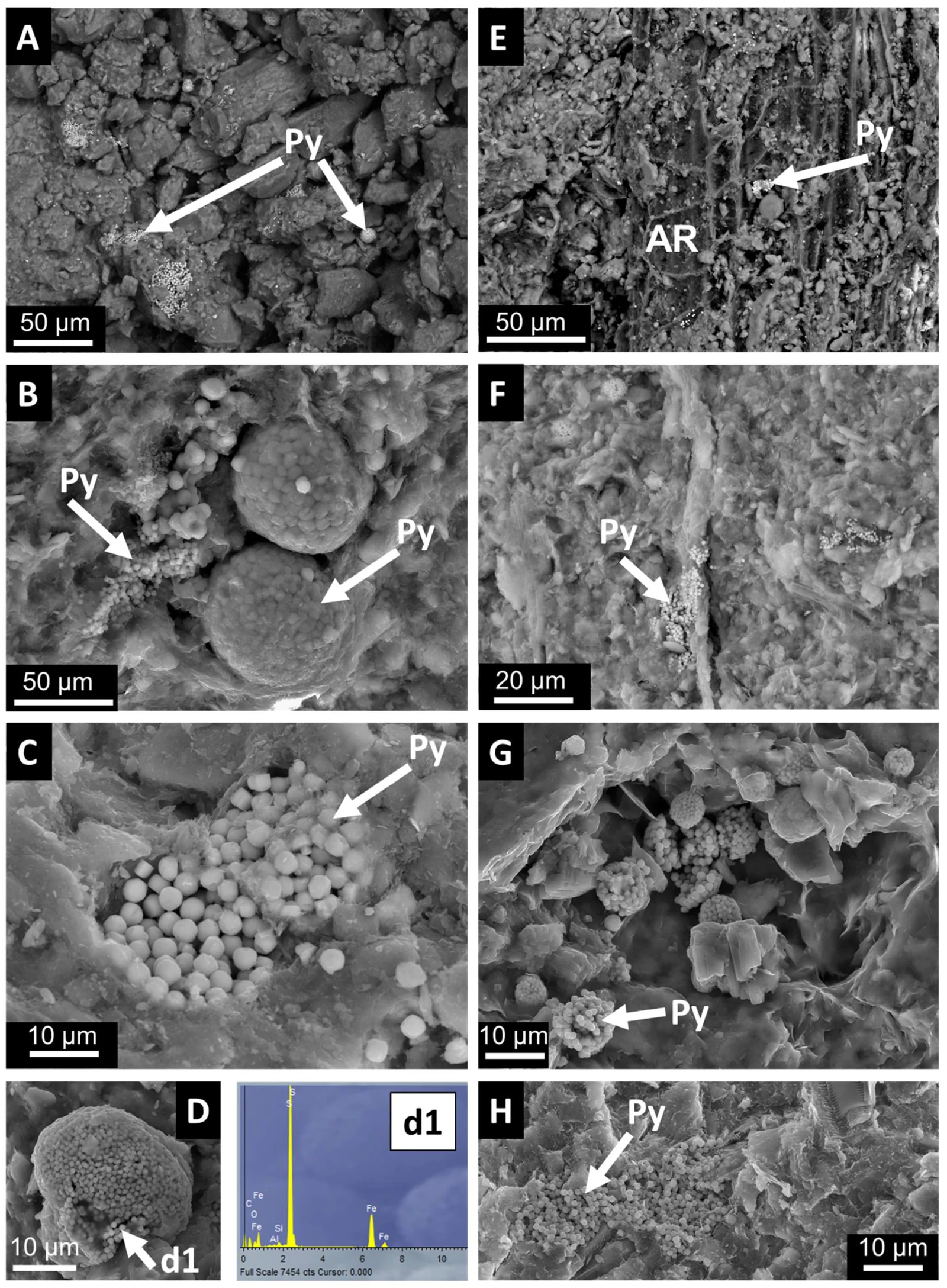
SEM images and EDX spectrum showing pyrite (Py)-dispersed microcrystals and framboids (**A**–**D**) appearing on algal remains (AR) (**E**) and infilling structures in plants (**F**–**H**).

**Figure 3:**
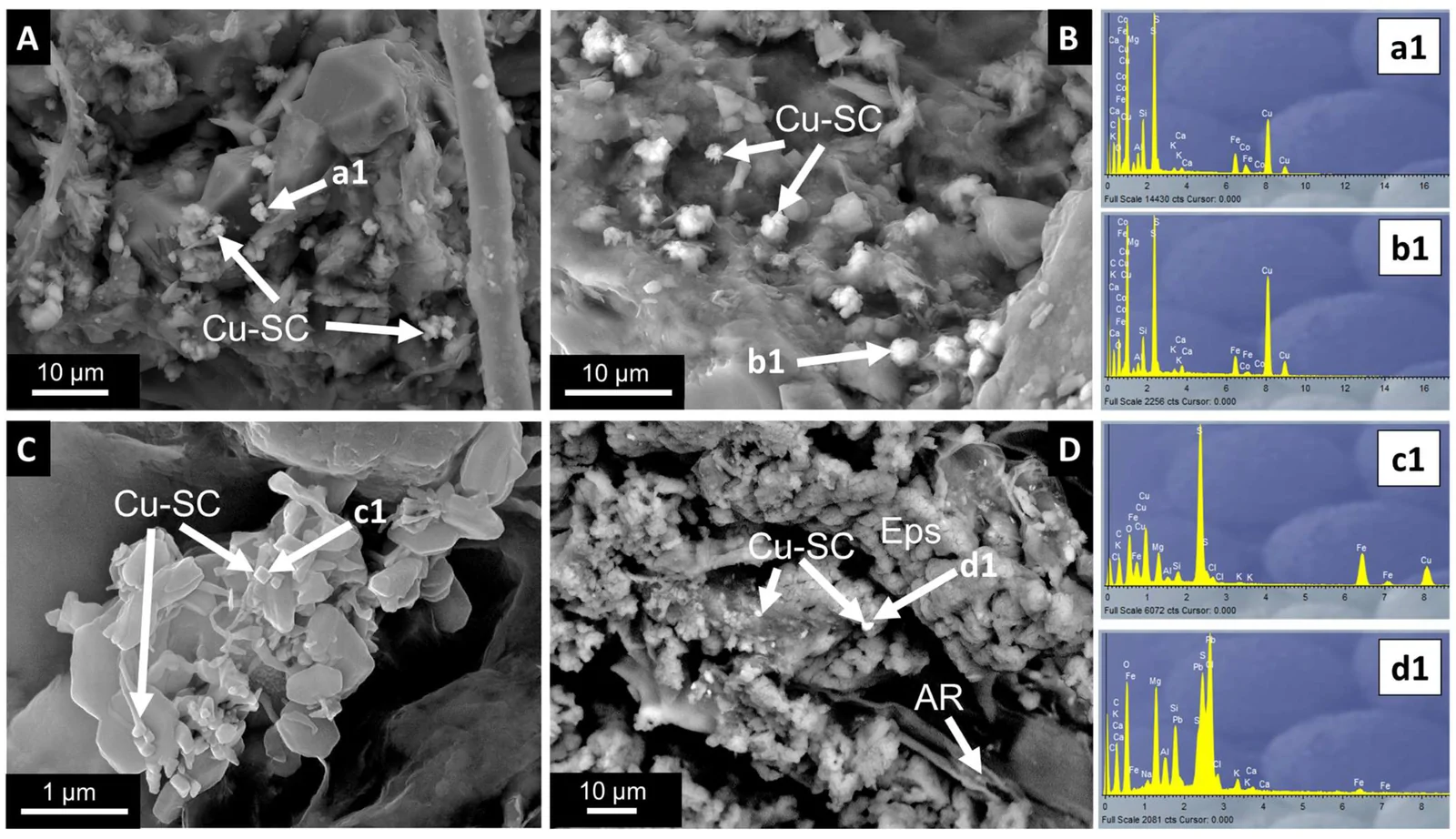
SEM images and EDX analyses showing sulfide crystals with variable Cu contents (Cu-SC) (**A**,**B**) developed on algal remains (ARs) with epsomite crusts (Eps) and microorganisms’ mineralized exudates (**C**,**D**).

Most sulfide crystals in Laguna Honda sediments were observed to be pyrite (Figure 2). SEM pictures revealed the presence of dispersed microcrystals (<1 μm) and pyrite framboids (up to 30 μm) composed of cubic nanocrystals with a honeycomb structure (Figure 2A–D). Pyrite crystals and framboids commonly appear on algal remains (Figure 2E) and the infillings of tubular structures in plants (Figure 2F–H). The use of a BSE detector and EDX analyses showed bright, frequent accumulations of small sulfide grains with variable Cu contents (Figure 3). Occasionally, Pb, Co, and Mn can be observed in the sulfide precipitates (Figure 3). These precipitates are commonly developed on algal surfaces and are surrounded by exudates, probably produced by microorganisms, which sometimes appear mineralized (Figure 3C).

### 3.2. HRTEM-EDX Characterization

**Figure 4:**
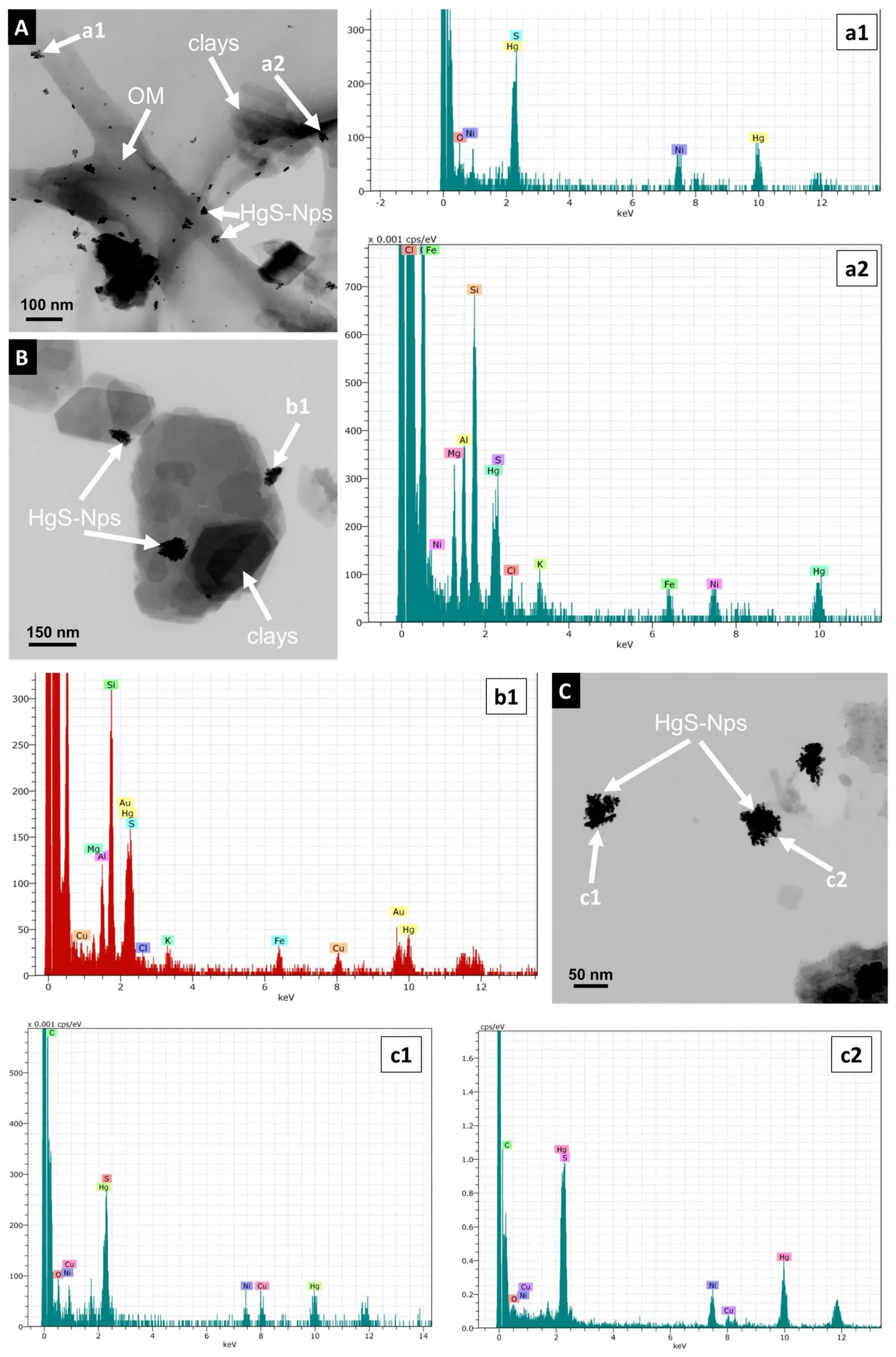
(**A**) HRTEM images showing HgS nanoparticles (HgS-Nps) adhered to organic matter and clay particles. (**B**) HgS-Nps on mica particles. (**C**) Isolated and dispersed HgS-Nps.

HRTEM images reveal the presence of metallic nanoparticles adhering to organic matter or clay particles (Figure 4A,B). The size of the metallic nanoparticles oscillates between 10 and 50 nm with a characteristic skeletal texture (Figure 4). AEM analysis of these particles revealed the presence of Hg and S. The spectra of particles that adhered to the clay grains showed significant peaks of Si, Al, Mg, and K, indicating mica contamination (Figure 4(a2)). AEM analyses from isolated nanoparticles showed significant Hg and S peaks without any influence of the clay matrix (Figure 4(c1,c2)). The Hg-S ratio in the spectra is consistent with that of HgS, although electron diffraction patterns for these HgS grains are rather poor. Cu peaks are systematically associated with the relationship between Hg and S. Some associations between nanoparticles are characterized by the presence of Au peaks, which decrease in intensity from the inner to the outer part of the nanoparticle association (Figure 4(b1)). Ni peaks in the spectra are produced by the grid.

### 3.3. Chemical Composition of the Sediments

Sediment composition in saline wetlands results from detrital and anthropic inputs and their interactions with the water table and communities of organisms in the ecosystem. Many saline wetlands at the eastern end of the Guadalquivir Basin become polluted due to agricultural practices of olive cultivation. A set of 130 samples was geochemically analyzed from 54 points of a sampling network in Laguna Honda.

Significant compositional differences in major elements (Table 2) and trace elements (Table 3) were observed between materials sampled near the wetland shore and those from areas closer to the center of the permanently flooded lagoon.

Shoreline sediments showed the highest contents of SiO_2_ (up to 54.42% in LH-81-3), Al_2_O_3_ (up to 14.98% in LH-20-3), Fe_2_O_3_ (up to 6.15% in LS-1A-R2), K_2_O (up to 4.50% in LS-1A-R2), and TiO_2_ (up to 0.75% in LS-1A-R2) (Table 2). Medina et al. [42,46] found significant amounts of quartz and illite in sediment samples from the shore of Laguna Honda. However, some samples from the center of the lake may have similar contents of Al_2_O_3_, Fe_2_O_3_, and K_2_O to those observed in the shore samples (e.g., LH-13-1).

The highest contents of LOI and CaO were found in some samples from the northern shore of the lagoon (e.g., LH-52-3 with 32.56% LOI and 31.1% CaO); however, samples from its central part, such as LH-67-5, showed the highest LOI values (36.13%), albeit slightly lower in CaO (12.58%). Samples from the eastern shore had the highest TOC contents (LHP-3, LHP-5, LS1-A, LS1A-RR, and LH-20-5, up to 3.65%). Some of these samples contain significant amounts of S (LHP-5, 8.84%), Cu (LS1A-RR, 47 ppm), and MgO (LH-20-5, 5.30%) (Table 2 and Table 3). The concentration of Cu in these sediments is clearly higher than the value of unpolluted reference sediments surrounding the wetland from the CSCs (Table 3). Moreover, this concentration is above the European mean Cu concentration in olive groves (33.5 ppm) [15], classifying this soil as exceeding the reference for Cu pollution in Spain (50 ppm) [53]. Some samples with a Cu content similar to that of LS1A-RR are characterized by low levels of Zr (for example, LH-54-5 with 66 ppm Zr). These compositional differences coincide with the mineralogical distribution, as indicated by Medina et al. [46], who revealed that samples rich in carbonates and sulfates were the poorest in silicates. The samples richest in Au (LHP-5 with 21.9 ppm and LH-65-3 with 14 ppm) are also located on the eastern shore, and these exhibit a very significant REE content (up to 158 ppm in LH-65-3). Medina et al. [42] revealed that the soil samples surrounding the wetland and sediments from the CSC where Laguna Honda formed have Au contents below the detection limit (Table 3). Some samples rich in Au and REE, such as LH 84-5 and LHP-5, are also characterized by the highest Hg values (up to 5 ppm). The Hg contents in these sediments exceed the standard reference for contaminated soils in Spain (1 ppm) [53] and are clearly higher than the value of unpolluted reference sediments surrounding the wetland from CSCs (Table 3).

These samples also have significant contents of TOC and S (up to 3.39% and 8.84%, respectively, in LHP-5). Medina et al. [42] revealed the presence of high Au contents in detrital phyllosilicate-rich samples from the lagoon shore.

## 4. Discussion

A comparison of Cu, Hg, and Au concentrations was performed in wetland sediments using chemical analyses of the geological substrate (Table 3) and the regulatory framework for contaminants in sediments and soils in Spain [53]. The results reveal that the background concentrations of these metals are very low, indicating that their concentrations in wetland sediments are not related to the regional background. As such, their presence must be due to pollution. Zapater et al. [54] related the presence of hazardous substances in olive oil and soils of olive groves to the large-scale application of chemical pesticides and fertilizers during the growing season. Charfi et al. [55] attributed high concentrations of Cu to preventive treatment with fungicides to combat olive peacock spot, such as copper oxychloride, copper hydroxide, and copper sulfate, applied at different periods throughout the year in olive groves from Jaén and Cordoba provinces in the Guadalquivir river basin. Casanova et al. [56] indicated that olive peacock spot affects 60% of olive groves; it is considered an endemic disease in the provinces of Jaén, Córdoba, and Sevilla in the Andalusia region. Copper fungicides are applied by thoroughly spraying the whole canopy of the tree with the fungicide solution. Regular annual treatments with relatively high doses of copper are necessary, leading to copper accumulation in olive soils [56]. Moreover, regarding Au accumulation in Laguna Honda sediments, Medina et al. [42] indicated that the sponge-like morphology of Au aggregates formed by chain-like fused nanoparticle structures observed in shore sediments from Laguna Honda is frequently observed in experiments where high concentrations of Au nanoparticles are added that can be easily condensed. Therefore, a reasonable hypothesis would be that the enrichment of Laguna Honda sediments in heavy metals is associated with the agricultural practices of the olive groves surrounding the wetland.

The relationship between detrital inputs, carbonaceous matter content, authigenic mineral crystallization, and trace element fixation processes was evaluated using statistical factor analysis. Four factors related to possible processes occurring in the lake were obtained, explaining 87.28% of the system variance (Table 4).

The first factor (41.77% of variance) explains two associations between elements. K, Fe, Al, Si, and Ti have high positive weights and were associated with a detrital source from the surrounding materials. Given that these elements are significant in the composition of illite, which is especially found in the mineralogical composition of shore lake sediments and are commonly considered detrital elements, e.g., [25,57], a detrital source linked to nearby CSC sediments from the Guadalquivir Basin for this association is proposed. Medina et al. [42] revealed that sediments from the shoreline of the Laguna Honda wetland were enriched in phyllosilicates from surrounding CSC material sediments, which avoided the precipitation of authigenic carbonates. Ca and LOI show negative weights in the first factor, which can be associated with calcium carbonate precipitation in the sediments. Medina et al. [46] revealed carbonate precipitation in sediments from Laguna Honda due to high water carbonate and bicarbonate concentrations, promoted by microbial organic matter oxidation, high-temperature CO_2_ degassing in water, and CO_2_ fixation due to algal photosynthesis.

For the second factor (24.02 of variance), Mg, Cu, organic matter content, and S had positive weights. This grouping is negatively correlated with depth and Zr content. Medina et al.’s study [46] revealed that mineralized algal crusts in Laguna Honda are characterized by the formation of a thick layer composed of poorly crystalline Mg-sulfate (epsomite) and halite, which are associated with evaporation and SOM activity. The precipitation of epsomite and halite is very common during the final steps of saline brine evaporation [58,59]. Therefore, the second factor is associated with the formation of floating algal mats that provide a high organic matter content in the shallowest sediments. Here, the formation of epsomite and halite crusts is prominent. Moreover, high organic matter concentrations lead to Cu adsorption from agricultural treatments. HRTEM images revealed the presence of Cu-containing metallic particles adhered to organic matter (Figure 4). Rosolen et al. [2] indicated that Cu and other elements, usually used as pesticides and macro- and micro-nutrients for plants, can be fixed in wetland organic matter, impacting element uptake and increasing their concentration in the upper parts of soils and sediments in association with organic matter [60]. The electron microscope images of sulfide nanoparticles enriched in Cu (Figure 3 and Figure 4) and the presence of SRM-enriched microbial community observed by Medina et al. [46] in Laguna Honda sediments indicate that SRM activity is an important factor regulating the transformation of adsorbed Cu in organic matter to crystallized sulfide minerals. These processes can also explain the significant amounts of Hg and S in the fourth factor of statistical treatment (10.21% of variance), suggesting that Hg is fixed through the precipitation of sulfides, mediated by the activity of SRM in the Laguna Honda sediments described in Medina et al.’s previous study [46]. Wolfenden et al. [61] indicated that in organic matter-rich sediments, mercury is fixed as a sulfide phase even in substantially oxic environments.

Finally, the third factor (11.28% of variance) is the relationship between accumulating Au and REE, suggesting that Au nanoparticles undergo a mechanical transport process from agricultural treatments. Medina et al. [42] indicated that inappropriate application of Au-bearing pesticides in the surrounding olive orchards resulted in collisions between Au nanoparticles as well as efficient coagulation and deposition of detrital Au-homoaggregates in gold-rich sediments at the shore of the lake and Au-illite heteroaggregates in sediments at the central part of the lake.

## 5. Conclusions

The Laguna Honda wetland (Guadalquivir Basin, southern Spain) acts as a significant environmental sink for heavy metals, specifically Cu, Hg, and Au, which originate from intensive Mediterranean olive cultivation, among other sources. The application of agrochemicals in the surrounding catchment area according to the agricultural practices described in the area [56] leads to anthropogenic metal enrichment in sediments.

Statistical analysis of the geochemical and mineralogical data reveals a spatial zoning and distinct partition between detrital inputs (silicates such as illite and quartz), particularly at the shoreline, and authigenic processes (carbonate and sulfide precipitation) concentrated in organic-rich, permanently flooded central areas.

The immobilization of trace elements, especially Cu and Hg, within the sediments is primarily driven by the authigenic precipitation of low-solubility sulfide minerals, including pyrite framboids and metallic nanoparticles of CuS and HgS. Medina et al.’s previous study [46] identified a significant SRM community in Laguna Honda sediments. Thus, sulfide-mediated sequestration is biogeochemically controlled by the crystallization of sulfide phases, promoted by SRM’s metabolic activity, which draws on the high availability of sulfate ions in hypersaline waters under the reducing conditions provided by the high TOC levels of sediments. This organic matter originates from floating algal mats developing on wetland water surfaces, which provide a critical substrate for initial metal adsorption. Mineral transformation and stabilization of pollutants are subsequently carried out.

The geochemical correlation between Au and REE indicates that both are governed by similar mechanical transport processes. The immobilization of Au is driven by the collision and rapid coagulation of nanoparticles, resulting in the sequestration of detrital Au-homoaggregates in phyllosilicate-rich shoreline sediments and Au-illite heteroaggregates in central areas of the wetland.

## Figures and Tables

**Table 1:** Water and sediment physicochemical properties of the Laguna Honda. PSU: practical salinity unit, ORP: oxidation and reduction potential. TOC: total organic carbon. Max: maximum; Min: minimum. Reproduced from Medina et al. [42] under the CC BY 4.0 license, 2024, MDPI.

		Mean	Min.	Max.
*Waters*				
Conductivity	mS/cm	71.62	70.88	73.00
Salinity	PSU	47.56	46.97	48.20
pH		9.40	9.20	9.55
Eh	mV	−132.54	−165.00	−111.00
ORP		−0.30	−215.00	150.50
HCO_3_^−^	mg/L	24.72	17.76	40.63
CO_3_^2−^	mg/L	67.20	58.01	70.42
Cl^−^	mg/L	21,633.12	21,370.29	22,203.93
NO_2_^−^	mg/L	2.52	0.00	12.59
Br^-^	mg/L	63.53	29.34	72.87
NO_3_^−^	mg/L	48.06	46.84	50.38
SO_4_^2−^	mg/L	24,537.05	24,142.58	24,840.72
Na^+^	mg/L	11,461.16	11,348.04	11,601.27
K^+^	mg/L	144.60	143.10	147.84
Ca^2+^	mg/L	1058.25	1016.64	1082.41
Mg^2+^	mg/L	8566.24	8489.00	8667.02
Sr^2+^	mg/L	22.52	21.07	24.78
*Sediments*				
TOC	mS/cm	1.03	0.17	2.25
Salinity	PSU	1.83	1.56	2.49
pH		7.54	6.33	8.54
Eh	mV	−26.26	−80.90	26.40
Conductivity	mS/cm	13.08	10.48	17.36
Chlorite	%	5	1	21
Illite	%	40	5	60
Quartz	%	21	7	45
Calcite	%	15	4	37
Gypsum	%	12	0	72
Dolomite	%	3	0	8
Aragonite	%	2	0	19
Halite	%	1	0	4
Pyrite	%	1	0	13

**Table 2:** Wetland sediment compositions of major elements determined by XRF (SiO_2_, Al_2_O_3_, Fe_2_O_3_, MnO, MgO, CaO, Na_2_O, K_2_O, TiO_2_, P_2_O_5_, S), loss of ignition (LOI), and total organic carbon (TOC) (as a weight percentage).

Sample	Depth	SiO_2_	Al_2_O_3_	Fe_2_O_3_	MnO	MgO	CaO	Na_2_O	K_2_O	TiO_2_	P_2_O_5_	S	TOC	LOI
LH-1.5	2.50	33.56	12.1	5.03	0.1	5.44	12.87	1.94	3.57	0.73	0.15	1.80	2.25	20.46
LH-1.3	13.5	53.14	12.78	5.44	0.06	4.56	6.83	0.6	3.92	0.64	0.13	0.19	0.83	10.82
LH-1.1	27.5	48.47	13.89	6.04	0.09	5.48	6.06	0.6	4.17	0.67	0.14	0.25	0.88	11.06
LH 2.5	2.50	39.28	14.81	5.88	0.07	5.7	10.57	0.82	4.2	0.67	0.12	1.92	2.98	16.85
LH 2.3	13.5	34.92	9.94	3.79	0.08	4.17	18.46	0.55	2.88	0.48	0.11	0.19	0.91	18.54
LH 2.1	27.5	41.93	13.47	5.22	0.06	5.08	12.35	0.47	3.86	0.67	0.12	0.23	1.04	16.33
LH-3.5	2.50	41.99	10.5	4.16	0.06	4.28	15.26	0.67	3.11	0.52	0.1	0.71	0.986	17.7
LH-3.3	13.5	32.56	10.11	3.95	0.08	4.34	18.6	0.75	2.95	0.45	0.12	3.24	0.402	17.84
LH-3.1	27.5	40.09	13.72	5.36	0.06	5.27	12.22	0.49	4.01	0.63	0.13	0.53	0.337	16.4
LH-5.5	2.50	39.1	14.18	5.64	0.06	5.27	11.43	0.79	4.06	0.59	0.12	1.38	0.652	16.78
LH-5.3	13.5	39.81	14.03	5.69	0.09	5.66	10.96	0.61	4.07	0.63	0.13	0.74	0.905	16.58
LH-5.1	27.5	40.44	14.44	5.73	0.06	5.52	11.25	0.59	4.21	0.62	0.13	0.48	0.173	16.19
LH-7.5	2.50	34.1	12.89	5.32	0.07	5.85	12.38	1.04	3.67	0.61	0.12	2.91	1.89	19.03
LH-7.3	13.5	36.33	12.74	5.04	0.09	5.29	13.21	0.73	3.62	0.58	0.12	2.31	0.797	17.9
LH-7.1	27.5	37.23	12.56	5.06	0.07	5.1	12.41	0.83	3.58	0.54	0.12	2.02	0.664	17.56
LH-9.5	2.50	34.64	12.92	5.26	0.1	5.51	12.53	1.06	3.71	0.53	0.12	2.24	1.35	18.25
LH-9.3	13.5	35.78	11.63	4.61	0.05	4.88	14.5	0.83	3.39	0.55	0.11	2.06	2.04	17.39
LH-9.1	27.5	36.06	9.72	3.98	0.05	4.13	16.33	0.8	2.77	0.45	0.1	3.87	0.708	16.07
LH 10.5	2.50	37.87	14.41	5.28	0.05	5.66	12.28	0.95	4.05	0.58	0.12	3.21	2.150	17.32
LH 10.3	13.5	37.4	14.46	5.52	0.08	5.35	11.65	0.66	4.04	0.59	0.12	0.87	1.340	16.88
LH 10.1	27.5	43.1	11.97	4.73	0.05	4.19	13.36	0.65	3.35	0.61	0.11	0.92	1.280	16.62
LH-11.5	2.50	36.8	13.44	5.24	0.07	5.31	12.45	1.01	3.82	0.55	0.11	1.86	1.28	18.07
LH-11.3	13.5	38.05	13.54	5.27	0.07	5.04	13.09	0.62	3.87	0.59	0.12	1.13	0.867	17.4
LH-11.1	27.5	38.97	13.39	5.47	0.08	5.18	12.59	0.74	3.77	0.59	0.13	1.01	1.02	17.24
LH 12.5	2.50	36.79	14.62	5.34	0.1	6.31	10.74	1.49	4.12	0.58	0.12	2.81	3.010	18.1
LH 12.3	13.5	32.63	12.51	4.97	0.06	5.32	13.65	0.93	3.47	0.53	0.1	1.18	1.41	19.32
LH 12.1	27.5	28.75	10.92	4.15	0.13	5.6	15.92	0.83	3	0.45	0.1	0.91	0.980	18.36
LH-13.5	2.50	30.57	11.12	4.35	0.1	5.5	15.54	1.52	3.15	0.48	0.12	4.36	1.62	18.33
LH-13.3	13.5	37.78	13.76	5.16	0.07	5.6	11.77	1.11	3.88	0.57	0.12	1.34	0.713	17.07
LH-13.1	27.5	38.91	14.98	5.76	0.06	5.69	10.23	0.88	4.23	0.6	0.13	0.97	0.987	17.05
LH-15.5	2.50	35.98	13.21	5.14	0.06	5.58	12.11	1.21	3.74	0.54	0.11	2.15	1.51	17.77
LH-15.3	13.5	36.93	13.14	5.05	0.08	5.44	12.4	0.99	3.72	0.55	0.12	1.76	0.954	17.09
LH-15.1	27.5	40.77	10.94	4.43	0.1	5.08	12.82	1.13	3.11	0.52	0.11	1.14	0.841	17.54
LH 16.5	2.50	37.12	13.69	5.29	0.07	6.04	11.45	1.25	3.8	0.57	0.12	1.14	1.420	18.14
LH 16.3	13.5	43.55	9.3	3.65	0.06	4.64	13.51	1.17	2.62	0.52	0.09	0.92	1.230	17.05
LH 16.1	27.5	39.23	12.08	4.41	0.03	5.55	11.17	1.56	3.34	0.57	0.11	1.09	1.250	17.51
LH 20.5	2.50	38	14.06	5.31	0.08	5.3	11.93	0.76	4	0.65	0.13	2.92	3.560	18.33
LH 20.3	13.5	39.19	14.66	5.81	0.06	5.19	11.66	0.57	4.15	0.63	0.13	2.78	3.030	16.76
LHP5	2.50	42.14	13.81	5.49	0.08	5.28	12.36	0.63	3.91	0.68	0.13	8.84	3.390	17.52
LHP3	13.5	38.76	13.46	5.31	0.06	4.93	12.53	0.6	3.8	0.64	0.13	3.03	3.650	18.09
LHP1	27.5	33.41	8.28	3.55	0.04	3.63	23.95	0.31	2.41	0.47	0.09	0.78	1.130	22.89
LS1A-ARR	2.50	46.3	13.84	5.76	0.1	5.45	7.04	0.87	4.15	0.06	0.15	5.01	3.500	14.58
LS1A-R2	13.5	53.35	14.85	6.15	0.08	5.07	3.56	0.55	4.5	0.75	0.14	4.22	3.330	10.03
LS1A-AB	27.5	52.36	11.87	5.06	0.08	4.38	7.81	0.44	3.61	0.58	0.11	1.17	1.400	10.4
LH 51.5	3	21.43	6.95	2.88	0.053	5.45	15.29	2.64	2	0.29	0.1	2.13	1.01	30.97
LH 51.3	24	32.24	8.04	3.23	0.042	3.75	22.11	1.02	2.31	0.40	0.1	1.43	0.51	26.5
LH 51.1	36	31.96	11.32	4.71	0.091	5.42	15.28	1.55	3.17	0.46	0.14	6.81	0.93	24.31
LH 52.5	3	27.17	10.08	3.86	0.065	5.54	16	1.86	2.77	0.39	0.12	3.05	1.51	28.52
LH 52.3	24	21.15	5.87	2.7	0.038	2.56	32.56	0.61	1.64	0.26	0.11	0.51	0.44	31.1
LH 53.5	3	27.9	10.15	4	0.073	7	12.24	3.36	2.81	0.42	0.09	2.70	0.52	29.8
LH 53.3	24	29.17	9.37	3.61	0.058	4.85	16.43	1.76	2.57	0.39	0.1	4.69	0.71	27.03
LH 53.1	36	33.6	12.02	4.72	0.07	5.6	11.99	1.31	3.39	0.49	0.12	3.25	0.63	23.77
LH 54.5	3	24.33	9.14	3.57	0.099	5.6	17.64	2.33	2.53	0.35	0.14	2.81	2.39	32.23
LH 54.3	24	27.89	7.83	3.19	0.063	3.9	24.47	0.95	2.17	0.35	0.13	0.94	1.24	28.72
LH 55.5	3	29.65	8.34	3.08	0.041	4.27	20.38	1.27	2.38	0.39	0.11	2.26	0.73	27.98
LH 55.3	24	22.44	7.85	3.74	0.106	4.63	18.9	1.21	2.23	0.32	0.12	6.43	1.44	27.7
LH 55.1	36	33.22	9.78	3.71	0.119	4.72	16.9	0.94	2.82	0.43	0.12	1.80	0.84	25.18
LH 56.5	3	28.56	9.97	4.03	0.062	6.93	10.39	2.93	2.88	0.43	0.11	3.61	0.5	29.61
LH 56.3	24	26.95	10.17	3.94	0.064	6.45	10.59	2.71	2.84	0.40	0.11	3.49	0.99	30.88
LH 57.5	3	35.4	12.34	4.62	0.073	5.46	12.64	1.25	3.42	0.51	0.14	1.45	0.89	23.21
LH 58.5	3	36.04	11.96	4.49	0.068	4.98	14.31	0.99	3.38	0.53	0.11	1.28	0.74	21.94
LH 58.3	24	38.16	12	4.6	0.061	5.01	14.63	0.75	3.44	0.54	0.13	0.64	0.49	20.87
LH 59.5	3	26.27	9.77	3.69	0.053	5.55	14.29	2.11	2.72	0.38	0.11	4.49	1.23	28.47
LH 59.3	24	36.88	11.27	4.49	0.056	5.03	14.28	0.93	3.22	0.51	0.1	1.32	0.55	21.61
LH 60.5	3	33.85	10.06	3.83	0.056	5.44	12.76	1.93	2.81	0.47	0.11	2.34	0.75	26.85
LH 60.3	24	31.92	11.36	4.4	0.061	5.41	13.77	1.65	3.19	0.47	0.12	3.24	0.59	25.4
LH 60.1	36	35.46	12.78	4.79	0.062	5.43	11.98	1.1	3.55	0.52	0.13	1.92	0.51	22.74
LH 61.5	3	38.52	12.08	4.64	0.054	5.46	11.93	1.15	3.37	0.52	0.13	0.97	0.56	21.61
LH 62.5	3	37.82	12.55	4.74	0.059	5.25	11.9	1.2	3.46	0.53	0.12	0.62	0.59	21.8
LH 62.3	24	37.54	12.91	4.97	0.082	5.24	12.94	0.79	3.69	0.54	0.13	0.91	0.61	21.17
LH 63.5	3	33.43	11.71	4.5	0.064	5.35	13.19	1.42	3.26	0.48	0.13	2.84	1.1	24.86
LH 63.3	24	37.67	11.35	4.29	0.055	4.62	15.04	0.82	3.16	0.5	0.11	1.41	0.49	21.26
LH 64.5	3	35.11	11.5	4.55	0.069	5.43	13.9	1.45	3.25	0.49	0.12	2.15	0.66	23.44
LH 64.3	24	37.4	14.01	5.21	0.072	5.73	10.37	1.02	3.88	0.54	0.13	1.23	0.47	21.52
LH 64.1	36	38.16	13.57	5.07	0.058	5.36	11.38	0.84	3.76	0.55	0.13	1.00	0.57	23.6
LH 65.5	3	47.67	9.35	3.78	0.057	3.8	10.28	1.1	2.74	0.50	0.09	1.13	0.68	21.26
LH 65.3	24	52.04	12.55	5.04	0.047	3.23	6.43	0.72	3.63	0.64	0.08	3.21	0.16	13.89
LH 66.5	3	39.13	9.38	3.43	0.062	4.49	12.37	1.58	2.72	0.45	0.09	2.09	0.47	24.32
LH 66.3	24	50.36	9.64	3.86	0.079	3.43	10.84	0.89	2.89	0.52	0.09	0.42	0.15	18.11
LH 66.1	36	48.18	10.44	4.01	0.055	3.31	10.54	0.87	3.04	0.55	0.09	1.17	0.19	19.48
LH 67.5	3	21.52	7.9	3.18	0.068	7.18	12.58	3.4	2.2	0.30	0.09	5.17	0.9	36.13
LH 67.3	24	29.56	11.03	4.03	0.069	6.24	10.79	2.44	2.98	0.42	0.1	3.59	0.76	30.46
LH 67.1	36	28.1	9.72	3.95	0.062	5.4	12.39	2.13	2.71	0.40	0.1	3.12	1.19	32.94
LH 68.5	3	30.45	10.85	4.74	0.201	5.16	15.12	1.17	3.09	0.42	0.15	1.89	2.41	27.47
LH 68.3	24	41.63	9.74	3.78	0.058	4.46	15.16	0.51	3.09	0.52	0.11	0.32	0.6	20.65
LH 69.5	3	29.06	10.89	4.3	0.075	5.6	14.84	1.88	3.07	0.43	0.1	4.54	0.92	27.96
LH 69.3	24	33.05	12.25	4.63	0.099	5.3	12.03	1.19	3.42	0.48	0.12	3.03	1.12	26.49
LH 70.5	3	36.42	11.54	4.48	0.07	5.15	11.31	1.26	3.28	0.48	0.11	4.47	0.62	24.56
LH 70.3	24	35.44	11.26	4.45	0.071	5.16	11.66	1.27	3.25	0.49	0.09	3.79	1.08	27.57
LH 70.1	36	32.24	11.93	4.55	0.087	6.11	10.54	1.35	3.38	0.49	0.12	6.10	1.43	29.31
LH 71.5	3	34.14	12.57	4.95	0.062	6.17	8.58	1.89	3.56	0.52	0.11	2.73	0.89	26.03
LH 71.3	24	41.88	11.09	4.55	0.113	4.86	11.22	0.81	3.3	0.52	0.13	4.38	0.66	21.93
LH 72.5	3	33.64	10.19	4.06	0.058	5.63	12.66	1.92	2.9	0.47	0.11	2.46	0.63	26.4
LH 72.3	24	38.27	13.15	5.25	0.066	5.24	9.9	0.95	3.78	0.58	0.12	1.23	0.54	22.47
LH 72.1	36	31.29	10.22	4.12	0.075	5.06	12.9	1	2.95	0.43	0.1	3.54	1.15	28.15
LH 73.5	3	33.37	11.32	4.6	0.063	5.88	10.8	1.89	3.33	0.49	0.11	2.31	0.62	26.6
LH 73.3	24	36.75	13.57	5.25	0.082	6.02	8.67	1.26	3.89	0.54	0.13	1.98	0.53	24.46
LH 73.1	36	36.55	12.25	4.91	0.065	5.45	10.51	1.28	3.49	0.54	0.12	2.31	0.76	25.08
LH 81.5	3	34.42	10.36	4.22	0.088	5.43	11.97	1.91	3.07	0.46	0.12	2.22	1.59	26.21
LH 81.3	24	54.42	10.49	4.28	0.074	4.65	5.22	1.05	3.16	0.60	0.11	3.51	0.18	14.36
LH 82.5	3	29.64	10.77	4.24	0.068	6.49	10.23	2.94	3.13	0.43	0.11	3.25	1.12	30.03
LH 82.3	24	42.19	10.78	4.45	0.058	5.2	11.11	1.03	3.14	0.53	0.11	1.13	0.37	22.21
LH 83.5	3	36.71	12.31	4.92	0.069	6.25	9.56	1.9	3.62	0.54	0.12	2.01	0.68	24.75
LH 83.3	24	37.72	13.63	5.52	0.076	6.37	7.64	1.55	3.97	0.57	0.13	1.88	0.76	23.63
LH 83.1	36	39.67	12.45	5.21	0.062	5.45	10.34	0.92	3.63	0.59	0.11	1.10	0.41	21.95
LH 84.5	3	37.19	12.77	5.17	0.078	5.56	9.41	1.18	3.89	0.56	0.13	2.07	1.1	24.5
LH 85.5	3	39.29	12.26	4.93	0.065	5.34	10.85	1.1	3.71	0.56	0.11	0.80	0.47	22.37
LH 86.5	3	33.32	12.36	4.83	0.078	6.17	10.2	1.97	3.53	0.49	0.12	2.63	0.83	26.34
LH 87.5	3	36.35	12.71	4.97	0.069	5.16	13.13	1.21	3.5	0.54	0.12	1.33	0.74	22.91
LH 88.5	3	35.06	12.53	4.82	0.07	5.53	11.55	1.55	3.51	0.51	0.13	1.55	0.76	24.93
LH 89.5	3	34.82	12.54	4.95	0.055	4.95	13.2	1.26	3.39	0.53	0.1	1.49	0.7	23.96
LH 89.3	24	35.2	12.9	5.06	0.092	5.52	11.91	1.34	3.63	0.51	0.11	1.63	0.76	23.17
LH 89.1	36	38.5	11.77	4.97	0.067	5.18	13	0.77	3.43	0.54	0.11	1.84	0.5	21.68
LH 90.5	3	36.34	13.54	5.12	0.061	5.22	11.13	0.94	3.76	0.54	0.13	1.53	0.93	23.52
LH 91.5	3	33.06	11.76	4.96	0.087	5.01	14.08	0.97	3.27	0.47	0.13	2.08	1.45	24.42
LH 92.5	3	30.58	11.49	4.47	0.088	5.74	13.56	2.13	3.21	0.46	0.13	2.36	1.13	27.73
LH 92.3	24	36.49	12.75	5.24	0.073	5.1	11.92	0.89	3.6	0.53	0.1	1.65	0.58	23.35
LH 92.1	30	40.67	13.15	5.34	0.08	4.9	11.41	0.52	3.66	0.59	0.11	0.46	0.62	20.34
LH 93.5	3	34.43	12.27	4.88	0.07	5.19	12.91	1.39	3.41	0.50	0.11	2.11	0.8	24.08
LH 93.3	24	39.05	14.03	5.23	0.063	4.84	11.69	0.71	3.84	0.57	0.14	0.49	0.44	20.35
LH 94.5	3	32.94	12.13	4.61	0.064	6.45	10.79	2.59	3.39	0.48	0.1	2.25	0.52	27.23
LH 94.3	24	38.33	13.4	5.39	0.055	5.02	11.31	0.89	3.71	0.56	0.11	0.63	0.43	22
LH 94.1	36	39.66	11.51	4.84	0.074	4.61	12.76	0.74	3.23	0.53	0.09	0.66	0.43	21.14
LH 95.5	3	29.39	10.88	4.18	0.068	6.42	11.43	2.61	3	0.43	0.1	3.37	0.63	29.56
LH 95.3	24	37.34	13.59	5.33	0.056	5.43	9.88	1.03	3.79	0.55	0.12	6.79	0.44	23.59
LH 96.5	3	29.31	11.06	4.55	0.072	6.96	7.89	2.6	3.14	0.43	0.11	3.63	0.67	31.8
LH 96.3	24	32.38	11.27	4.55	0.057	6.11	11.02	2.31	3.2	0.47	0.11	3.31	0.57	27.92
LH 96.1	36	33.83	10.07	3.98	0.078	4.94	14.51	1.4	2.83	0.44	0.1	3.53	0.84	25.73
LH 97.5	3	30.04	12.06	4.69	0.074	5.74	10.95	2.11	3.25	0.44	0.1	3.79	0.7	29.9
LH 97.3	24	43.29	10.74	4.18	0.054	4.25	12.19	0.89	3.02	0.52	0.1	0.61	0.45	21.55

**Table 3:** Trace element sediment compositions determined by ICP-MS (in ppm). REE: Sum of the rare earth elements analyzed. LH, LS, and LHP: wetland sediments; CSC: substrate sediments. * Data for gold are derived from Medina et al. [42].

Sample	Depth	REE	Hg	Cu	Au	Zr
LH-1.5	2.50	-	2	38	2.31 *	81
LH-1.3	13.5	-	1	17	1.43 *	175
LH-1.1	27.5	-	1	18	0.63 *	159
LH 2.5	2.50	51.63	1	42.24	0.11	108.9
LH 2.3	13.5	47.32	0.44	19.49	0.00	150.3
LH 2.1	27.5	50.74	0.38	22.93	0.01	134.1
LH-3.5	2.50	-	1	32	1.89 *	140
LH-3.3	13.5	-	2.2	19	1.00 *	98
LH-3.1	27.5	-	1	24	0.37 *	113
LH-5.5	2.50	-	1.3	42	1.65 *	100
LH-5.3	13.5	-	1	27	1.02 *	108
LH-5.1	27.5	-	1	24	0.43 *	110
LH-7.5	2.50	-	2	26	0.97 *	82
LH-7.3	13.5	-	2.1	21	0.75 *	98
LH-7.1	27.5	-	1.5	20	0.32 *	107
LH-9.5	2.50	47.07	0.25	35.79	0.72 *	87
LH-9.3	13.5	45.37	1.1	24.04	0.68 *	100
LH-9.1	27.5	50.46	0.24	23.45	0.23 *	120
LH 10.5	2.50	49.82	2	36.73	0.00	110.4
LH 10.3	13.5	48.83	1	25.53	0.00	98.8
LH 10.1	27.5	53.89	1	23.7	0.08	154.2
LH-11.5	2.50	-	1.5	32	0.79 *	93
LH-11.3	13.5	-	1	26	0.81 *	101
LH-11.1	27.5	-	1	26	0.25 *	109
LH 12.5	2.50	47.85	2.5	33.56	0.00	101
LH 12.3	13.5	46.45	1	24.08	0.04	89.3
LH 12.1	27.5	43.60	1	18.6	0.16	96.5
LH-13.5	2.50	-	3	31	0.72 *	85
LH-13.3	13.5	-	1	33	0.69 *	106
LH-13.1	27.5	-	1	29	0.19 *	92
LH-15.5	2.50	-	1.1	31	1.45 *	89
LH-15.3	13.5	-	1	24	1.12 *	97
LH-15.1	27.5	-	1	22	0.43 *	147
LH 16.5	2.50	59.11	0.04	25.48	0.00	106.3
LH 16.3	13.5	54.07	0.17	20.08	0.00	169.3
LH 16.1	27.5	49.37	1	21.32	0.00	128.3
LH 20.5	2.50	48.99	2	37.51	0.00	104.3
LH 20.3	13.5	56.81	2	32.83	0.00	103.9
LHP5	2.50	111.24	4	35.89	21.90 *	108.7
LHP3	13.5	112.57	3	38.13	2.41 *	110.3
LHP1	27.5	78.79	1	18.98	0.47	106.8
LS1A-ARR	2.50	135.18	1	47.45	1.30	150.1
LS1A-R2	13.5	137.14	3	43.58	1.40	165.9
LS1A-AB	27.5	117.38	1	23.73	1.23	174.8
LH 51.5	3	71.85	1	34	1.00	61
LH 51.3	24	94.04	2	21	4.00	116
LH 51.1	36	119.90	1	25	1.00	90
LH 52.5	3	104.42	1	67	1.00	75
LH 52.3	24	71.51	1	17	1.00	74
LH 53.5	3	103.40	1	32	1.00	79
LH 53.3	24	100.40	1	22	1.00	88
LH 53.1	36	133.25	1	25	1.00	92
LH 54.5	3	89.10	1	47	1.00	66
LH 54.3	24	89.30	1	23	1.00	95
LH 55.5	3	95.73	1	24	1.00	99
LH 55.3	24	79.94	3	22	6.00	65
LH 55.1	36	108.93	1	24	1.00	110
LH 56.5	3	101.24	1	39	1.00	80
LH 56.3	24	97.02	1	28	1.00	71
LH 57.5	3	125.01	1	46	1.00	97
LH 58.5	3	128.15	1	40	1.00	109
LH 58.3	24	125.59	2	30	2.00	118
LH 59.5	3	96.03	1	35	1.00	71
LH 59.3	24	118.58	1	29	1.00	119
LH 60.5	3	106.06	1	28	1.00	117
LH 60.3	24	113.43	1	33	1.00	89
LH 60.1	36	126.42	1	26	1.00	97
LH 61.5	3	124.17	1	43	1.00	106
LH 62.5	3	125.37	1	42	1.00	105
LH 62.3	24	131.22	1	42	1.00	101
LH 63.5	3	118.47	1	42	1.00	91
LH 63.3	24	121.90	1	32	1.00	112
LH 64.5	3	118.13	1	39	1.00	100
LH 64.3	24	134.81	3	32	8.00	94
LH 64.1	36	136.28	1	38	1.00	100
LH 65.5	3	119.05	1	19	1.00	211
LH 65.3	24	147.29	1	13	14.00	175
LH 66.5	3	105.45	1	18	1.00	160
LH 66.3	24	111.14	1	11	12.00	175
LH 66.1	36	120.31	1	37	1.00	169
LH 67.5	3	78.48	1	21	1.00	60
LH 67.3	24	104.71	1	20	1.00	78
LH 67.1	36	96.82	1	21	1.00	77
LH 68.5	3	111.83	2	39	5.00	81
LH 68.3	24	116.25	1	22	1.00	148
LH 69.5	3	108.15	1	35	1.00	78
LH 69.3	24	120.18	1	47	1.00	89
LH 70.5	3	111.53	1	24	1.00	105
LH 70.3	24	119.67	1	20	1.00	106
LH 70.1	36	117.66	1	23	1.00	87
LH 71.5	3	125.72	3	48	5.00	93
LH 71.3	24	125.47	1	37	1.00	150
LH 72.5	3	107.13	1	31	1.00	103
LH 72.3	24	137.46	3	32	2.00	108
LH 72.1	36	105.43	1	20	1.00	92
LH 73.5	3	117.81	1	29	1.00	95
LH 73.3	24	135.99	1	27	1.00	97
LH 73.1	36	125.52	1	26	1.00	107
LH 81.5	3	109.77	1	47	1.00	109
LH 81.3	24	128.25	2	32	10.00	309
LH 82.5	3	110.86	1	39	1.00	81
LH 82.3	24	119.31	1	25	4.00	155
LH 83.5	3	120.04	4	45	6.00	110
LH 83.3	24	132.79	1	34	1.00	103
LH 83.1	36	125.95	1	27	1.00	121
LH 84.5	3	129.11	5	46	11.00	99
LH 85.5	3	120.23	1	42	1.00	114
LH 86.5	3	113.67	1	45	1.00	89
LH 87.5	3	118.26	2	42	3.00	118
LH 88.5	3	117.15	1	40	1.00	96
LH 89.5	3	115.63	1	37	1.00	99
LH 89.3	24	120.40	1	39	1.00	93
LH 89.1	36	123.77	1	29	1.00	104
LH 90.5	3	126.85	1	49	1.00	95
LH 91.5	3	107.60	1	44	2.00	89
LH 92.5	3	105.46	1	40	1.00	80
LH 92.3	24	127.90	2	35	3.00	95
LH 92.1	30	130.54	2	30	4.00	117
LH 93.5	3	110.71	1	39	1.00	95
LH 93.3	24	133.75	1	31	1.00	105
LH 94.5	3	113.68	1	33	1.00	87
LH 94.3	24	128.14	2	30	5.00	100
LH 94.1	36	122.33	1	40	1.00	124
LH 95.5	3	101.97	2	35	4.00	78
LH 95.3	24	133.08	1	30	1.00	95
LH 96.5	3	106.45	1	27	1.00	76
LH 96.3	24	108.71	2	33	2.00	92
LH 96.1	36	97.35	1	22	1.00	112
LH 97.5	3	103.24	1	26	1.00	75
LH 97.3	24	114.98	3	25	8.00	155
CSC-1	-	127	0.02	15	<0.001 *	123
CSC-2	-	131	0.02	17	<0.001 *	134

**Table 4:** Variable loadings in the factor analysis.

	F1	F2	F3	F4
K_2_O	0.94	0.31	−0.12	−0.10
Fe_2_O_3_	0.90	0.32	−0.12	−0.06
Al_2_O_3_	0.88	0.36	−0.16	−0.12
SiO_2_	0.80	−0.48	0.25	−0.09
TiO_2_	0.78	−0.09	−0.02	−0.06
LOI	−0.80	0.38	0.20	−0.15
CaO	−0.63	−0.30	−0.38	0.41
Na_2_O	−0.60	0.57	0.25	−0.30
MgO	−0.01	0.85	0.01	−0.34
Cu	0.13	0.54	0.07	0.10
TOC	0.23	0.57	−0.22	0.58
S	−0.22	0.50	0.27	0.42
Depth	0.08	−0.50	0.00	−0.17
Zr	0.42	−0.62	0.29	−0.04
Au	0.17	−0.07	0.72	0.38
REE	0.17	0.10	0.49	−0.21
Hg	0.23	0.24	0.42	0.46
MnO	0.08	0.28	−0.02	0.24
P_2_O_5_	0.41	0.40	−0.18	0.26
Variance%	41.77	24.02	11.28	10.21

LOI: Loss of ignition; REE: Sum of the rare earth elements analyzed.

## Data Availability

The original contributions presented in this study are included in the article. Further inquiries can be directed to the corresponding author.
